# Sample size calculation while controlling false discovery rate for differential expression analysis with RNA-sequencing experiments

**DOI:** 10.1186/s12859-016-0994-9

**Published:** 2016-03-31

**Authors:** Ran Bi, Peng Liu

**Affiliations:** Department of Statistics, Iowa State University, Snedecor Hall, Ames, Iowa, 50011 USA

**Keywords:** RNA-seq, FDR, Experimental design, Sample size calculation, Power analysis

## Abstract

**Background:**

RNA-Sequencing (RNA-seq) experiments have been popularly applied to transcriptome studies in recent years. Such experiments are still relatively costly. As a result, RNA-seq experiments often employ a small number of replicates. Power analysis and sample size calculation are challenging in the context of differential expression analysis with RNA-seq data. One challenge is that there are no closed-form formulae to calculate power for the popularly applied tests for differential expression analysis. In addition, false discovery rate (FDR), instead of family-wise type I error rate, is controlled for the multiple testing error in RNA-seq data analysis. So far, there are very few proposals on sample size calculation for RNA-seq experiments.

**Results:**

In this paper, we propose a procedure for sample size calculation while controlling FDR for RNA-seq experimental design. Our procedure is based on the weighted linear model analysis facilitated by the *voom* method which has been shown to have competitive performance in terms of power and FDR control for RNA-seq differential expression analysis. We derive a method that approximates the average power across the differentially expressed genes, and then calculate the sample size to achieve a desired average power while controlling FDR. Simulation results demonstrate that the actual power of several popularly applied tests for differential expression is achieved and is close to the desired power for RNA-seq data with sample size calculated based on our method.

**Conclusions:**

Our proposed method provides an efficient algorithm to calculate sample size while controlling FDR for RNA-seq experimental design. We also provide an R package *ssizeRNA* that implements our proposed method and can be downloaded from the Comprehensive R Archive Network (http://cran.r-project.org).

**Electronic supplementary material:**

The online version of this article (doi:10.1186/s12859-016-0994-9) contains supplementary material, which is available to authorized users.

## Background

During the past decade, next generation sequencing (NGS) technology has revolutionized genomic studies, and tremendous development has been made in terms of throughput, scalability, speed and sequencing cost. RNA-Sequencing (RNA-seq), also called Whole Transcriptome Shotgun Sequencing (WTSS), is a technology that uses the capabilities of NGS to study the entire transcriptome. Compared with microarray technologies that used to be the major tool for transcriptome studies, RNA-seq technologies have several advantages including a larger dynamic range of expression levels, less noise, higher throughput, and more power to detect gene fusions, single nucleotide variants and novel transcripts. Hence, RNA-seq technologies have been popularly applied in transcriptomic studies.

In a typical RNA-seq experiment, messenger RNA (mRNA) molecules are extracted from samples, fragmented, and reverse transcribed to double-stranded complementary DNA (cDNA). The cDNA fragments are then sequenced on a high-throughput platform, such as HiSeq by Illumina or SOLiD by Applied Biosystems. After sequencing, millions of DNA fragment sequences, called reads, are recorded and aligned to a reference genome. The number of reads mapped to each gene measures the expression level for that gene. Thus, RNA-seq provides discrete count data serving as measurements of mRNA expression levels, which is different from the fluorescence intensity measurements from microarray technologies that have been considered as continuous variables after transformation. As a result of high frequency of low integers, the statistical methods developed for analyzing microarray data are not directly applicable for RNA-seq data.

In the statistical analysis of RNA-seq data, identifying differentially expressed (DE) genes across treatments or conditions is a major step or main focus. A gene is considered to be DE across treatments or conditions if the mean read counts differ across treatment groups. Otherwise, we say the gene is equivalently expressed (EE). Many statistical methods have been proposed for the detection of DE genes with RNA-seq data. Some popular methods, including *edgeR* [1–4], *DESeq* [5] and *DESeq2* [6], are based on the negative binomial (NB) distribution. *QuasiSeq* [7] presented quasi-likelihood methods with shrunken dispersion estimates. A more recently proposed method by the Smyth group [8] works with log-transformed count data and captures the mean-variance relationship of the log-count data through a precision weight for each observation (using a function called *voom* in their R package) and then applies the *limma* method [9] for differential expression analysis.

Due to the genetic complexity and high-dimensionality of the resulting datasets, RNA-seq experiments require complicated bioinformatic and statistical analysis in addition to the cost of experimental materials and sequencing. Many experiments only employ a small number of replicates, in which cases the power of statistical inference is limited. However, if the sample size is too large (which is rare), it is also a waste of experimental materials and manpower. For these reasons, one of the principal questions in designing an RNA-seq experiment is: how many biological replicates should be used to achieve a desired power? In other words, how large of the sample size do we need?

To answer this question, we need to determine a sample size that is required to achieve a desired power while controlling an appropriate error rate. When calculating sample size for a single test, type I error rate is commonly used. Fang and Cui [10] discussed a sample size formula for a single gene based on likelihood ratio test or Wald test. Hart et al. [11] and their associated R package *RNASeqPower* [12] proposed a sample size calculation method for any single gene based on score test while controlling type I error rate. However, for RNA-seq data analysis, tens of thousands of genes are simultaneously tested for differential expression, which requires the correction of multiple testing error, and false discovery rate (FDR) [13] has been the choice of error criterion in RNA-seq data analysis.

Several sample size calculation methods while controlling FDR have been proposed in microarray experiments. For example, Liu and Hwang [14] developed a method to calculate sample size given a desired power and a controlled level of FDR by finding the rejection region for the test procedure and hence power for each sample size. Hereafter, we call this sample size calculation method the LH method. Orr and Liu [15] assembled the *ssize.fdr* R package which implements the LH method.

However, sample size calculation for RNA-seq data analysis while controlling FDR is underdeveloped. Some earlier studies performed sample size and power estimation for RNA-seq experiments under Poisson distribution [16–18], but the additional biological variation across RNA-seq samples yields overdispersion, which means the equal mean-variance relationship for the Poisson distribution does not adapt to the variability present in RNA-seq data. To account for overdispersion, the negative binomial distribution is more flexible to use. Li et al. [19] proposed a sample size determination method while controlling FDR based on the exact test implemented in *edgeR* that tests for genes differentially expressed between two treatments or conditions. This method calculates a sample size based on the minimum fold change of DE genes, the minimum average read counts of DE genes in the control group, and the maximum dispersion of DE genes under negative binomial models. As expected, such a method would be very conservative and not practically informative. The *RnaSeqSampleSize* R package [20] provides an estimation of sample size based on single read count and dispersion which implements Li et al.’s method. Also, instead of using the minimum average read counts and the maximum dispersion, *RnaSeqSampleSize* gives an estimation of sample size based on the read count and dispersion distributions estimated from real data, together with the minimum fold change, which is much better than Li et al.’s method, but would still be conservative due to the usage of the minimum fold change. The LH method is applicable as long as we can compute the power and type I error rate given a rejection region. However, there are no closed-form formulae for power for the popularly applied NB based methods. Then we have to rely on a lot of simulation to figure out quantities such as power and type I error rate for each sample size and each simulation setting [10]. Ching et al. [21] provided a power analysis tool that calculates the power for a given budget constraint for each size of samples, and then determined the sample size for a desired power. Wu et al. [22] introduced the concepts of stratified targeted power and false discovery cost, and estimated sample size by the evaluation of statistical power over a range of sample sizes based on simulation studies. Both Ching et al. and Wu et al.’s methods are simulation-based, thus we need to do a lot of simulations for power assessment for each sample size, which is time-consuming.

In this paper, we propose a much less computationally intensive method, which only demands one-time simulation, for sample size calculation in designing RNA-seq experiments. First, we use the *voom* method to model the mean-variance relationship of the log-count data of RNA-seq and produce a precision weight for each observation. Second, based on the normalized log-counts and associated precision weights, we estimate the distribution of weighted residual standard deviation of expression levels. Then for two-sample experiments, we derive a formula of the *t* test statistic in the weighted least squares setting and estimate the distribution of effect sizes for differential expression. Next, we apply the LH method to calculate the required sample size for a given desired power and a controlled FDR level. Our simulation demonstrates that the desired power is reached for data with the sample size calculated from our method for several popular tests for differential expression.

The article is organized as follows. The ‘Methods’ section describes our proposed method illustrated with the two-sample *t*-test. In the ‘Results and discussion’ section, we present four simulation studies based on either negative binomial distributions or real RNA-seq dataset, and our method provide reliable sample sizes for all simulation studies. The ‘Conclusions’ section discusses our results and some future work.

## Methods

In this section, we first review the *voom* method [8] and the LH method of sample size calculation. Then, we introduce our approach for calculating sample size while controlling FDR in designing RNA-seq experiments.

### The *voom* method

Suppose that an RNA-seq experiment includes a total of *N* samples. Each sample has been sequenced, and the resulting reads are aligned with a reference genome. The number of reads mapped to each reference gene is recorded. The RNA-seq data then consist of a matrix of read counts *r*_*gij*_, where *g*=1,2,…,*G* denotes gene *g*, *i*=1,2 denotes group where *i*=1 is for the control group and *i*=2 is for the treatment group, and *j*=1,2,…,*n*_*i*_ denotes replicates in each group with *N*=*n*_1_+*n*_2_. The idea of the *voom* method proposed by Law et al. [8] is to use precision weights to account for the mean-variance relationship and apply weighted least square analysis to RNA-seq data.

The method of *voom* starts from transforming the RNA-seq count data to the log-counts per million (log-cpm) value calculated by

$$ y_{gij} = {log}_{2} \left(\frac{r_{gij}+0.5}{R_{ij}+1} \times 10^{6} \right), $$

where $R_{ij} = \sum _{g=1}^{G} r_{gij} $ is the library size for the *i*th treatment and *j*th replicate. As has been done in [9], Law et al. then fit a linear model to the transformed data according to the experimental design. For each gene *g*, the following linear model

$$ \mathbf{y}_{g} = X \boldsymbol{\beta}_{g} + \boldsymbol{\varepsilon}_{g} $$

is fitted to $\mathbf {y}_{g} = (y_{g11}, \dots, y_{g1n_{1}}, y_{g21}, \dots, y_{g2n_{2}})'$, the vector of log-cpm values, where *X* is the design matrix with rows $\mathbf {x}_{ij}^{T}$, ***β***_*g*_ is a vector of parameters that may be parameterized to include *log*_2_-fold changes between experimental conditions, and ***ε***_*g*_ is the error term with *E*(***ε***_*g*_)=**0**.

Assuming that $ E(y_{gij}) = \mu _{gij} = \mathbf {x}_{ij}^{T} \boldsymbol {\beta }_{g} $, then by ordinary least squares, the above linear model is fitted for each gene *g*, which yields regression coefficient estimates $\boldsymbol {\hat {\beta }}_{g}$, fitted values $\hat {\mu }_{gij} = \mathbf {x}_{ij}^{T} \boldsymbol {\hat {\beta }}_{g} $, residual standard deviations *η*_*g*_ and fitted *log*_2_-read counts

$$ \hat{l}_{gij} = \hat{\mu}_{gij} +{log}_{2} (R_{ij}+1) - {log}_{2}(10^{6}). $$

To obtain a smooth mean-variance trend, Law et al. fit a LOWESS curve to the square root of residual standard deviations $\eta _{g}^{1/2}$ as a function of average log-counts $\tilde {r}_{g}$, where $ \tilde {r}_{g} = \bar {y}_{g} +{log}_{2}(\tilde {R} + 1) - {log}_{2}(10^{6}) $ with $\bar {y}_{g}$ being the average log-cpm value for each gene *g* and $\tilde {R}$ being the geometric mean of library sizes. Then for each observation *y*_*gij*_, the predicted square root residual standard deviation $\hat {\eta }_{gij}^{1/2}$ is obtained to be the LOWESS fitted value corresponding to $\hat {l}_{gij}$.

Finally, the *voom* precision weights are defined as the inverse variances $ w_{gij} = \frac {1}{\hat {\eta }_{gij}^{2}}$. Law et al. recommended analyzing the log-cpm data with weighted least squares, and the weights (*w*_*gij*_) are used to account for the mean-variance relationship in the log-cpm values. Assuming normal distribution for residual errors (***ε***_*g*_), methods such as *t*-tests or moderated *t*-tests can then be applied for differential expression analysis.

### The LH method of sample size calculation

In genomic studies, we simultaneously test a large number of hypotheses, each relating to a gene. Hence, multiple testing is commonly used in the analysis. Assume there are *G* genes in total and each gene is tested for the significance of differential expression. Table 1 summarizes the various outcomes that occur when testing *G* hypotheses, where *V* is the number of false positives, *R* is the number of rejections among the *G* tests, and *π*_0_ is the proportion of non-differentially expressed genes.

**Table 1:** Outcomes when testing *G* hypotheses

	Accept null	Reject null	Total
True nulls	*U*	*V*	*π*_0_ *G*
False nulls	*T*	*S*	(1−*π*_0_)*G*
Total	*W*	*R*	*G*

False discovery rate (FDR), defined by Benjamini and Hochberg [13], is the expected proportion of false positives among the rejected hypothesis:

$$ FDR = E \left(\left. \frac{V}{R} \right| R >0 \right) Pr(R > 0), $$

while positive FDR (pFDR), proposed by Storey [23], is defined to be

$$ pFDR = E \left(\left. \frac{V}{R} \right| R >0 \right). $$

Both FDR and pFDR are widely used error rates to control in multiple testing encounted in genomic studies. In RNA-seq experiments, most often we end up detecting DE genes, i.e. *R*>0. Hence, in this paper, we do not differentiate between FDR and pFDR.

Liu and Hwang [14] proposed a method for a quick sample size calculation for microarray experiments while controlling FDR. Let *H*=0 represent no differential expression (null hypothesis is true) and *H*=1 represent differential expression (null hypothesis is false). Based on the definition of pFDR and assumptions in [23] (all tests are identical, independent and Bernoulli distributed with *Pr*(*H*=0)=*π*_0_, where *π*_0_ is the proportion of EE genes), they derived that

(1) $$ \frac{\alpha}{1-\alpha} \frac{1-\pi_{0}}{\pi_{0}} \geq \frac{Pr(T \in \Gamma | H = 0)}{Pr(T \in \Gamma | H = 1)},  $$

where *α* is the controlled level of FDR, *T* denotes the test statistic and *Γ* denotes the rejection region of the test. Then for each comparison, the LH method calculates the sample size as follows. First, for a fixed proportion of non-differentially expressed genes, *π*_0_, and the level of FDR to control, *α*, they find a rejection region *Γ* that satisfies (1) for each sample size. Then for the selected rejection region *Γ* for each sample size, the power is calculated by *Pr*(*T*∈*Γ*|*H*=1). According to the desired power, a sample size is determined.

The rejection region depends on the test applied for differential expression, and the method based on (1) can be applied to any multiple testing procedure where the same rejection region is used. This LH method can be implemented using an R package, *ssize.fdr*, developed by Orr and Liu [15], and applied for designing one-sample, two-sample, or multi-sample microarray experiments. The method would be applicable to RNA-seq experiments if we can calculate power and type I error rate given a rejection region.

### Proposed method for RNA-seq experiments with two-sample comparison

For the popularly applied tests in RNA-seq differential expression analysis such as *edgeR* and *DESeq*, there are no closed-form expressions to calculate the two quantities *Pr*(*T*∈*Γ*|*H*=0) and *Pr*(*T*∈*Γ*|*H*=1). Hence, the LH method cannot be directly applicable to these methods. However, the recently proposed *voom* and *limma* analysis for RNA-seq data [8, 24] is based on weighted linear models and we can obtain tractable formulae for power and type I error rate. In this paper, our idea is to derive formulae to calculate power and type I error rate based on *voom* and weighted linear model analysis, and then apply the LH method for sample size calculation. We will use two-sample *t*-tests to illustrate our idea. Similar methods can be derived for other designs such as paired-sample or multiple treatments comparison.

Suppose our interest is to identify the differentially expressed (DE) genes between a treatment and a control group. Assuming that for gene *g*, group *i* and replicates *j*, we observe the RNA-seq data read counts *r*_*gij*_, where the mean for gene *g* in group *i* is *λ*_*gij*_=*d*_*ij*_*γ*_*gi*_. Here, *d*_*ij*_ stands for a normalization factor or effective library size that adjusts the sequencing depth for sample *j* in group *i*, *γ*_*gi*_ stands for the normalized mean expression level of gene *g* in group *i*. Then for each gene *g*, to test for differential expression means to test the hypothesis:

$$ {H_{0}^{g}}: \gamma_{g1} = \gamma_{g2} \hspace{3mm} vs. \hspace{3mm} {H_{1}^{g}}: \gamma_{g1} \neq \gamma_{g2}. $$

As reviewed in the first part of the ‘Methods’ section, when applying the *voom* method, the RNA-seq read counts *r*_*gij*_ are transformed to log-cpm values *y*_*gij*_ with associated weights *w*_*gij*_ and mean *μ*_*gi*_ for each sample *j* in group *i*. With this parameterization, testing for DE means testing

$$ {H_{0}^{g}}: \mu_{g1} = \mu_{g2} \hspace{3mm} vs. \hspace{3mm} {H_{1}^{g}}: \mu_{g1} \neq \mu_{g2}, $$

where *μ*_*g*1_ and *μ*_*g*2_ are the expectation of log-cpm values of *g*th gene for control and treatment group, respectively.

For each individual gene *g*, the weighted linear model

$$ \mathbf{y}_{g} = X \boldsymbol{\beta}_{g} + \sigma_{g} W_{g}^{-\frac{1}{2}} \boldsymbol{\epsilon} $$

can be fitted to log-cpm values

$$\mathbf{y}_{g} = \left(y_{g11}, \dots, y_{g1n_{1}}, y_{g21}, \dots, y_{g2n_{2}}\right) $$

with design matrix

$$X = \left(\begin{array} {cc} 1 & 0 \\ \vdots & \vdots \\ 1 & 0 \\ 1 & 1 \\ \vdots & \vdots \\ 1 & 1 \end{array} \right), $$

coefficients vector

$$\boldsymbol{\beta}_{g} = \left(\begin{array}{c} \beta_{g1} \\ \beta_{g2} \end{array} \right), $$

unknown gene-specific standard deviation *σ*_*g*_, and associated *voom* precision weights

$$W_{g} = diag(w_{g11}, \cdots, w_{g1n_{1}}, w_{g21}, \cdots, w_{g2n_{2}}). $$

Assuming $\boldsymbol {\epsilon } \sim MVN(\boldsymbol {0},I_{n_{1}+n_{2}})$, where MVN stands for multivariate normal distribution, the *t*-test statistic for gene *g* is

(2) $$ T_{g} = \frac{\hat{\beta}_{g2}}{S.E.(\hat{\beta}_{g2})},  $$

where the estimated *log*_2_-fold change between treatment and control group $\hat {\beta }_{g2}$ and its standard error $S.E.(\hat {\beta }_{g2})$ could be obtained through weighted least squares estimation.

To make the *t*-test based method more straightforward to apply, we reparameterize the formula (2) to

(3) $$ T_{g} = \frac{\Delta_{g}}{s_{g} \sqrt{\frac{1}{n_{1}} + \frac{1}{n_{2}}}},  $$

where

$$s_{g} = \sqrt{\frac{\left(\mathbf{y}_{g} - X \boldsymbol{\beta}_{g}\right)' W_{g} \left(\mathbf{y}_{g} - X \boldsymbol{\beta}_{g}\right)}{n_{1}+n_{2}-2}} $$

can be viewed as the pooled sample standard deviation, which is an estimator of *σ*_*g*_, and

(4) $$ \Delta_{g} \equiv \hat{\beta}_{g2} \sqrt{\frac{\bar{w}_{g1\cdot} \bar{w}_{g2\cdot} }{\bar{w}_{g\cdot \cdot}}}  $$

can be viewed as the scaled effect size which is defined by weighted mean difference of log-cpm values. Here, $\bar {w}_{g1\cdot } = \frac {1}{n_{1}} \sum _{j=1}^{n_{1}} w_{g1j}$, $\bar {w}_{g2\cdot } = \frac {1}{n_{2}} \sum _{j=1}^{n_{2}} w_{g2j}$ and $\bar {w}_{g\cdot \cdot } = \frac {1}{n_{1}+n_{2}} \sum _{i=1}^{2} \sum _{j=1}^{n_{i}} w_{gij}$. Details of the derivation for (3) is provided in the Appendix A.

After generating the effect size *Δ*_*g*_, and the standard deviation *σ*_*g*_ for each gene *g*, we could assume, as in [14], that the effect size follows a normal distribution

$$ \Delta_{g} \sim N\left(\mu_{\Delta}, \sigma_{\Delta}^{2}\right), $$

and the variance of log-cpm values for each gene follows an inverse gamma distribution

$$ {\sigma_{g}^{2}} \sim Inv-Gamma(a,b) $$

with mean $\frac {b}{a-1}$. Then we apply the LH method to calculate the optimal sample size given desired power and controlled FDR level. See Appendix B for a brief review of the calculations in the LH method involving in choosing the rejection region *Γ* safisfying formula (1).

Our proposed method requires the estimation of hyperparameters *μ*_*Δ*_, *σ*_*Δ*_, *a*, and *b*. If a relatively large pilot dataset is available, these parameters can be estimated based on the pilot data. Otherwise, we can simulate data to obtain the values for these hyperparameters. It has been shown that the NB model fits real RNA-seq data well [5]. In addition, many popularly applied tests for differential expression analysis of RNA-seq data are based on NB models. Hence, we suggest to simulate data according to NB models, and then use such simulated data to obtain the estimates of *μ*_*Δ*_, *σ*_*Δ*_, *a*, and *b*, which are then used to calculate sample size. We outline our proposed procedure for sample size calculation as follows:

1. For a given RNA-seq experiment, specify the following parameters:
  *G*: total number of genes for testing;
  *π*_0_: proportion of non-DE genes;
  *α*: FDR level to control;
  *pow*: desired average power to achieve;
  *λ*_*g*_: average read count for gene *g*=1,…,*G* in control group (without loss of generality, we assume that the normalization factors *d*_*ij*_ are equal to 1 for all samples);
  *ϕ*_*g*_: dispersion parameter for gene *g*;
  *δ*_*g*_: fold change for gene *g*.
  Note that *λ*_*g*_ and *ϕ*_*g*_ could be estimated from real data using methods such as *edgeR*.
2. Simulate RNA-seq read count data from a NB distribution with given parameters in step 1.
3. Use the *voom* and *limma* method to obtain the log-cpm value and the associated precision weight for each count, and then estimate effect size *Δ*_*g*_ according to (4) for each gene *g* and parameters *a*, *b* for the prior of *σ*_*g*_.
4. Estimate *μ*_*Δ*_ and *σ*_*Δ*_ by fitting
  $$ \Delta_{g} \sim N\left(\mu_{\Delta}, \sigma_{\Delta}^{2}\right). $$
5. Use the LH method to determine the sample size *n* to achieve desired power and controlled FDR level.

## Results and discussion

In this section, we present four simulation studies to evaluate our proposed method for sample size calculation for RNA-seq experiments. In the first three simulation studies, we set the total number of genes to be *G*=10,000 and the desired average power to be 80 %. The last simulation is real data-based.

### Simulation 1. Same set of parameters

We start from the simplest simulation setting where all genes share the same set of parameters for the NB distribution. Although such cases are unrealistic, they allow the method of Li et al. [19] to perform best because this method uses a single set of NB parameters (mean, dispersion, fold change) when calculating sample size. Hence, we use this simulation setting to study the performance of our method and compare it to the method of Li et al. We refer to the parameter settings from Table 1 in [19], and compare the resulting sample size and power calculated by both Li et al.’s method and our proposed method.

In the main manuscript, we present results for one of those parameter settings as an example: the proportion of non-DE genes *π*_0_=0.99, the mean read counts for control group *λ*=5 with normalization factors *d*_*ij*_=1, dispersion parameter *ϕ*=0.1, FDR controlling at level 0.05, and fold change *δ*=2 for differentially expressed genes. Suppose *r*_*gij*_ denotes the read count for gene *g*, group *i* and replicate *j*=1,2,…,*n*_*i*_ in each group with *n*_1_=*n*_2_=*n*. Then, for EE genes, both *r*_*g*1*j*_ and *r*_*g*2*j*_ were drawn from *NB*(5,0.1); for DE genes, *r*_*g*1*j*_ were drawn from *NB*(5,0.1) and *r*_*g*2*j*_ were drawn from *NB*(10,0.1) or *NB*(2.5,0.1).

**Fig. 1:**
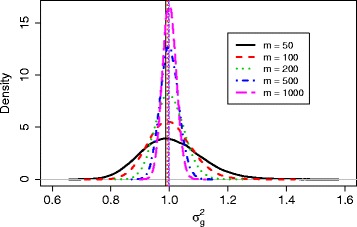
Fitted inverse gamma distributions of ${\sigma _{g}^{2}}$ for sample size *m*= 50, 100, 200, 500, 1000 for simulation 1

After setting these simulation parameters in step 1, we follow steps 2-4 to simulate data and obtain the values of hyperparameters. To investigate the effect of this simulation step, we tried different sizes of simulated data, *m*=50,100,200,500,1000, where *m* is the sample size for each group in step 2 of our procedure. For each *m*, we generated read counts *r*_*g*1*j*_ (control group) and *r*_*g*2*j*_ (treatment group) from independent NB distributions for every gene *g* and sample *j*, *g*=1,…,*G*, *j*=1,…,*m*. After using *voom* and *lmFit* in the R package *limma* [9] to produce weights *w*_*gij*_ for each observation, we then obtained effect size *Δ*_*g*_ for each gene and parameters *a*, *b* for the prior distribution of ${\sigma _{g}^{2}}$. The fitted inverse gamma distributions of ${\sigma _{g}^{2}}$ for each *m* are shown as in Fig. 1, with vertical lines indicating the modes. It seems that the mode doesn’t change much, and the distribution of ${\sigma _{g}^{2}}$ shrinks towards the center as sample size gets larger.

After obtaining the fitted parameters, we calculated sample size according to our proposed method described in the third part of the ‘Methods’ section to achieve a desired power of 80 %. We then simulated data according to each calculated sample size and checked whether the desired power was achieved. In Table 2, the first three columns listed our simulation results corresponding to this simulation setting. As *m* increased from 50 to 100 to 1000, the calculated sample size dropped from 35 to 34 and 32, respectively. This decrease is expected because the parameters were estimated more precisely with larger *m*. For example, the distribution of ${\sigma _{g}^{2}}$ shrank as *m* increased as shown in Fig. 1. The effect on the resulting sample size is not big, at most with a difference of 3 (35 vs. 32).

**Table 2:** Sample size and anticipated average power calculated by our method and observed average power by the *voom* and *limma* pipeline while controlling FDR using *q*-value procedure based on parameters at different *m* for three simulation settings

	Simulation 1			Simulation 2					Simulation 3		
	Our Method			Our Method			*RnaSeqSampleSize*		Our Method		
m	Sample	Anticipated	Observed	Sample	Anticipated	Observed	Sample	Estimated	Sample	Anticipated	Observed
	size	power	power	size	power	power	size	power	size	power	power
50	35	0.802	0.858	13	0.810	0.876	9	0.780	22	0.800	0.801
100	34	0.814	0.846	13	0.814	0.876	9	0.724	22	0.803	0.801
200	34	0.815	0.846	13	0.823	0.876	9	0.765	22	0.804	0.801
500	33	0.817	0.833	13	0.827	0.876	9	0.769	22	0.805	0.801
1000	32	0.800	0.804	13	0.826	0.876	9	0.764	22	0.805	0.801

We also present the comparison between our method and *RnaSeqSampleSize* R package for simulation 2, where the right two columns are sample size and power calculated by the *RnaSeqSampleSize* R package

**Fig. 2:**
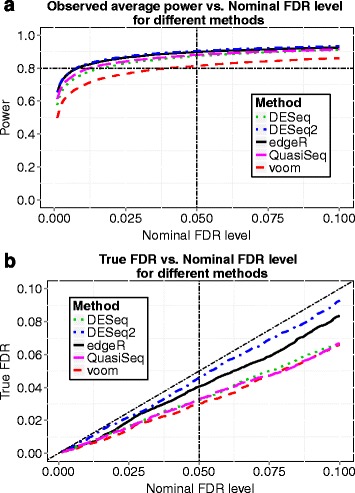
Results from simulation 1. Data were simulated with sample size *n*=32. **a** Observed average power from different methods of differential expression analysis is plotted against the nominal FDR level controlled using the *q*-value procedure. **b** The actual FDR level versus the nominal FDR level for different methods

**Fig. 3:**
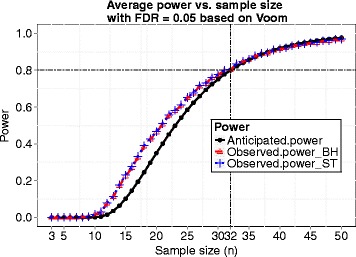
Anticipated power curve calculated by *ssizeRNA* and observed power curves using *voom* and *limma* while FDR was controlled using either the Benjamini and Hochberg method (BH) or the *q*-value procedure by Storey and Tibshirani (ST) for simulation 1

We now choose a sample size *n*=32 and demonstrate this sample size indeed reaches the desired power 0.8. At *n*=32, we simulated 100 datasets and performed several popularly applied tests such as the *edgeR* exact test, the *voom* and *limma* method, *DESeq*, *DESeq2* and *QuasiSeq* using the corresponding R packages. Desired power (0.8) was achieved for all testing methods when controlling FDR at 0.05 using *q*-value procedure [25], and the observed FDR was controlled successfully under all the five methods. The results are shown in Fig. 2. For the *voom* and *limma* pipeline method, the observed power curves while FDR was controlled using the Benjamini and Hochberg’s method [13] and the *q*-value procedure [25] and the power curve based on our calculation are shown in Fig. 3. The observed power was obtained by averaging actual power over 100 simulated datasets for each sample size. The observed power and the power calculated by our method are close with our calculation being a little conservative. Hence, our proposed method provides an accurate estimate of power, and the sample size calculated by our method is reliable.

**Fig. 4:**
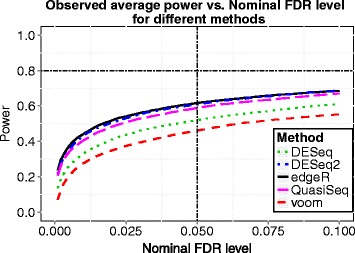
Observed average power vs. nominal FDR for five methods at sample size *n*=20 calculated by Li et al.’s method for simulation 1

Finally we would like to compare our method with other existing sample size calculation methods, including Li et al.’s approach [19, 20] and Wu et al.’s approach [22]. Li et al. proposed to calculate the sample size by “using a common $\rho ^{*} = {argmin}_{g \in M_{1}} \{|{log}_{2}(\rho _{g})|\}$ minimum fold change”, where *ρ*_*g*_ in their paper denotes the fold change and is equivalent to *δ*_*g*_ in this paper. However, we found that the direction of fold change does matter when applying their code. If we set *ρ*_*g*_=2, the sample size calculated by their method is *n*=20, as presented in their Table 1. The plot of average power vs. nominal FDR for their method is shown in Fig. 4, from which we notice that the desired power (0.8) is not achieved at sample size *n*=20 when controlling FDR at 0.05. In fact, the observed power is 0.6166 when using the *edgeR* exact test based on which they derived their method. When applying the the *voom* and *limma* pipeline, the observed power is 0.4608 for sample size 20. If we set *ρ*_*g*_=0.5, then the sample size will be 32, same as our proposed method, and we get power of 0.8988 using the *edgeR* exact test and 0.8149 using the *voom* and *limma* pipeline for differential expression analysis. Wu et al. (*PROPER*) provided a simulation-based power evaluation tool, which requires a lot of simulations to assess the power for each sample size. Table 3 presents the computation time needed for the calculation. It took *PROPER* 6.5 hours to get the resulting sample size while the other two methods only needed seconds. *PROPER* is more than 1,300 fold time-consuming than our proposed method. The resulting sample size from *PROPER* is 25, less than our proposed method. This is because *PROPER* is based on *edgeR* exact test, which tends to be more powerful than the *voom* and *limma* pipeline.

**Table 3:** Comparison of sample size calculation methods, including the proposed method in this paper, Zhao et al.’s approach (*RnaSeqSampleSize*) and Wu et al.’s approach (*PROPER*).

	Simulation 1			Simulation 2			Simulation 3		
Method	Sample	Power	Computation	Sample	Power	Computation	Sample	Power	Computation
	size		time	size		time	size		time
Our method	34	0.815	17.3 sec	13	0.823	16.8 sec	22	0.804	15.5 sec
*RnaSeqSampleSize*	32	0.810	0.3 sec	9	0.765	54.6 sec	74	0.742	82.8 sec
*PROPER*	25	0.806	6.5 h	10	0.804	1.5 h	19	0.805	3.5 h

Results determined by our method were based on parameters estimated at *m*=200. Power was evaluated based on the *voom* and *limma* pipeline for our method, while *edgeR* for *RnaSeqSampleSize* and *PROPER*. The computation time for each simulation was calculated on a MacAir laptop with 1.3 GHz i7 CPU and 4GB RAM

Results for other parameter settings under *m*=200 are presented in the Additional file 1, with Li et al.’s results in the first row, and our results in the second row.

### Simulation 2. Gene-specific mean and dispersion with fixed fold change

In the second simulation setting, we used a real RNA-seq dataset to generate gene-specific mean and dispersion parameters. A maize dataset was obtained from a study by Tausta et al. [26], who compared gene expression between bundle sheath and mesophyll cells of corn plants.

Similar to simulation 1, we generated 10,000 genes from *NB*(*λ*_*g*_,*ϕ*_*g*_), with fold change *δ*=2 for DE genes, *λ*_*g*_ and *ϕ*_*g*_ from the means and dispersions estimated for each gene in the maize dataset. For EE genes, both *r*_*g*1*j*_ and *r*_*g*2*j*_ were drawn from *NB*(*λ*_*g*_,*ϕ*_*g*_); for DE genes, *r*_*g*1*j*_ were drawn from *NB*(*λ*_*g*_,*ϕ*_*g*_) and *r*_*g*2*j*_ were drawn from *NB*(2*λ*_*g*_,*ϕ*_*g*_) or *NB*(0.5*λ*_*g*_,*ϕ*_*g*_). The proportion of non-DE genes was *π*_0_=0.8.

**Fig. 5:**
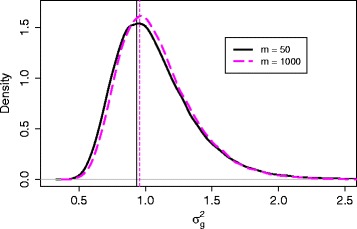
Fitted inverse gamma distributions of ${\sigma _{g}^{2}}$ for sample size *m*= 50 and 1000 for simulation 2

The fitted inverse gamma distributions of ${\sigma _{g}^{2}}$ for *m*= 50 and 1000 are very similar, as shown as in Fig. 5, where vertical lines indicate the modes. The middle three columns in Table 2 give the sample size and average power calculated by our *ssizeRNA* package. As shown in Table 2, the resulting sample sizes are all 13 when *m* ranges from 50 to 1000. This is expected because Fig. 5 indicates that the estimated distributions of ${\sigma _{g}^{2}}$ are very close using different *m* values for this dataset.

**Fig. 6:**
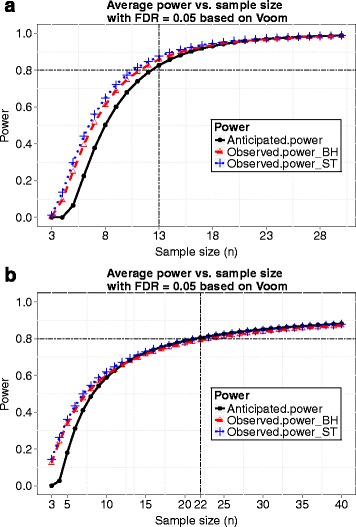
Anticipated power curve calculated by *ssizeRNA* and observed power curves using *voom* and *limma* while FDR was controlled using either the Benjamini and Hochberg method (BH) or the *q*-value procedure by Storey and Tibshirani (ST) for simulation 2 (in (**a**)) and 3 (in (**b**))

At *n*=13, we checked the plots of average power vs. nominal FDR and true FDR vs. nominal FDR, and the results were similar to those obtained in simulation 1. More specifically, the desired power (0.8) was achieved, and FDR was controlled successfully. Actually, the desired power can be reached at sample size *n*=11. Figure 6(a) gives the power curve calculated by our method based on hyperparameters estimated at *m*=1000 together with observed power curves with FDR controlled by the Benjamini and Hochberg’s method and the *q*-value procedure, respectively. The anticipated power curve based on *m*=1000 is close to the other two observed power curves.

The *RnaSeqSampleSize* R package [20] could give an estimation of sample size and power by prior real data. They first use user-specified number of genes to estimate the gene read count and dispersion distribution, then *sample_size_distribution* and *est_power_distribution* functions will be used to determine sample size and actual power. When we used the same real dataset as our simulation setting 2, the sample size calculated by their method was 7, with actual power 0.774, which did not reach the desired power 0.8. We also tried to apply their method using our simulated data (with different *m*), the resulting sample size is larger (*n*=9). The power estimated by their method at *n*=9 are shown in Table 2, and all their estimated power were actually smaller than 0.8. *PROPER* started from an estimation of mean and dispersion parameters, which is similar to our method. The sample size calculated by their method is 10, with power 0.804 based on DE detection method *edgeR*. The comparison results of our proposed method and these three approaches are shown in the middle three columns of Table 3. Still, *PROPER* is much more time-consuming than the other two methods.

### Simulation 3. Gene-specific mean and dispersion with different fold change

In this simulation, the setting is the same as the second simulation study, except that the fold change *δ*_*g*_ was simulated from a log-normal distribution for differentially expressed genes. For EE genes, both *r*_*g*1*j*_ and *r*_*g*2*j*_ were drawn from *NB*(*λ*_*g*_,*ϕ*_*g*_); for DE genes, *r*_*g*1*j*_ were drawn from *NB*(*λ*_*g*_,*ϕ*_*g*_) and *r*_*g*2*j*_ were drawn from *NB*(*λ*_*g*_*δ*_*g*_,*ϕ*_*g*_) or *NB*(*λ*_*g*_/*δ*_*g*_,*ϕ*_*g*_) where

$$ \delta_{g} \sim log-normal(log(2), 0.5log(2)). $$

The last three columns in Table 2 give the sample size and power calculated by our method. As in simulation 2, varying the size of simulated data (*m*) did not result in different sample sizes. Anticipated and observed power curves are presented in Fig. 6(b), from which we notice that the three curves are almost indistinguishable after power reaches 60 %. This more realistic simulation demonstrates that our proposed method provides accurate power and sample size.

We also applied *RnaSeqSampleSize* to this simulation setting. Since their method is based on minimum fold change, such results will be conservative due to the variability of fold change, especially as in this case, the minimum fold change is close to 1. When we used the 10th percentile of fold change of DE genes as the “minimum” fold change, the sample size calculated by their method was 74, which is still much larger than what we actually need, but the power calculated by their method based on the “minimum” fold change was less than the desired power 0.8. *PROPER* gave a result of sample size 19 with power 0.805 based on DE detection method *edgeR*. The comparison results of our proposed method and these two approaches are shown in the last three columns of Table 3.

Based on results from simulations, our proposed method and *RnaSeqSampleSize* provided answers much faster than *PROPER*, and our proposed method and *PROPER* provided good sample size estimation. Overall, our proposed method worked the best while both accuracy and computation time are considered.

### Simulation 4. Real data-based simulation

Our method involves simulating data based on negative binomial distributions. To check the robustness of our method, we conducted a simulation based on a real RNA-seq dataset from [27], which was upon an RNA-seq experiment that sequenced 69 lymphoblastoid cell lines (LCL) derived from unrelated Nigerian individuals. We used the genes with minimum read counts across all individuals larger than 10, which results in 9154 genes. First, we estimated the mean and dispersion across all 69 individuals for each gene. Assume that fold change comes from a log-normal distribution as in simulation 3,

$$ \delta_{g} \sim log-normal(log(2), 0.5log(2)), $$

the proportion of non-DE genes being 80 %, to reach a desired power 0.8 while controlling FDR at 0.05, the sample size calculated by our method is 12 at *m*=200.

**Fig. 7:**
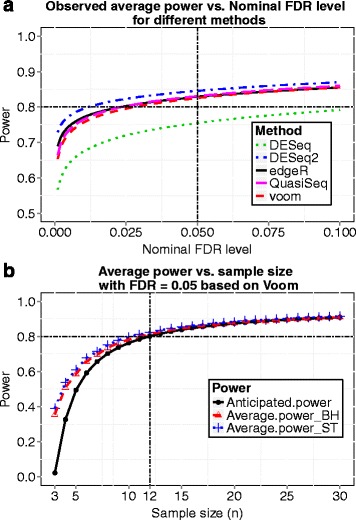
Results from simulation 4. **a** Observed average power from different methods of differential expression analysis is plotted against the nominal FDR level controlled using the *q*-value procedure at sample size *n*=12. **b** Anticipated power curve calculated by *ssizeRNA* and observed power curves using *voom* and *limma* while FDR was controlled using either the Benjamini and Hochberg method (BH) or the *q*-value procedure by Storey and Tibshirani (ST)

To check whether desired power can be achieved at the calculated sample size, we simulated 100 datasets. For each simulation, we randomly picked 24 out of the 69 individuals and randomly assigned 12 individuals to the control group and the remaining 12 individuals to the treatment group. Consider all 9154 genes among the 24 individuals as EE since the samples were randomly selected from the same population. Then we randomly generated 20 % of the 9154 genes to be DE, and their counts in the treatment group were multiplied by fold change *δ*_*g*_ which were drawn from a *log*−*normal*(*log*(2),0.5*log*(2)) distribution. The scaled counts were rounded to the nearest integers. This strategy likely results in more realistic data because all counts come from real dataset and no distributional assumptions were imposed. The plot of average power vs. nominal FDR at *n*=12 is shown in Fig. 7(a), where desired power (0.8) was achieved for most testing methods, including *edgeR*, *DESeq2*, *QuasiSeq*, *voom* and *limma* methods, when controlling FDR at 0.05 using *q*-value procedure. Figure 7(b) gives the power calculated by our method based on hyperparameters estimated at *m*=200. It also presents the observed average power curves when FDR was controlled by either the Benjamini and Hochberg’s method or the *q*-value procedure. The anticipated power curve based on *m*=200 is close to the other two observed power curves. Hence, our proposed method also provides a reliable estimation of sample size and power in the most realistic simulation study.

## Conclusions

In recent years, RNA-seq technology has become a major platform to study gene expression. With large sample size, RNA-seq experiments would be rather costly; while insufficient sample size may result in unreliable statistical inference. Thus sample size calculation is a crucial issue when designing an RNA-seq experiment. Although we could use a lot of simulations for each sample size and determine the one that reach our desired power as suggested in [10, 21, 22], this requires generous calculation and lacks efficiency. Our method provides a quick calculation for sample size, which only demands one-time simulation. From the simulation studies in the section of Results and discussion, we demonstrate that our proposed method offers a reliable approach for sample size calculation for RNA-seq experiments.

For each gene *g*, when we use a two-sample *t*-test to do differential expression analysis, the effect size *Δ*_*g*_ in formula (4) depends on the simulated sample size *m*. Larger *m* may lead to better estimation of the prior distributions and hence a more accurate sample size. Based on our simulation studies, the effect of *m* on the resulting sample size is not big, and *m*=200 should be enough for providing a relatively precise sample size.

The ordinary t-test instead of the moderated *t*-test [9] was used in *ssizeRNA* R package. Because the ordinary *t*-test is a bit less powerful than the moderated *t*-test, it tends to overestimate the sample size which might be the reason why our calculated sample size in simulation 2 is a little bit larger than what we actually need according to the observed power curves using *voom* and *limma*. However, the overestimation is not dramatic and far less than the method of Li et al. [19].

In this article, we illustrate our idea using a method for two-sample comparison with the *t*-test, because detecting differentially expressed genes between two treatment groups is the most common case in RNA-seq analysis. Our idea could be applied to multi-sample comparison with an F-test or tests for linear contrasts of treatment means as well.

The R package *ssizeRNA* implements our proposed sample size calculation method for RNA-seq experiments and it is freely available on the Comprehensive R Archive Network (http://cran.r-project.org). To install this package, start R and enter:

■■■ source(“http://bioconductor.org/biocLite.R”)
■■■ biocLite(“ssizeRNA”)

## Appendices

### Appendix A: Derivation of Eq. (3)

For each individual gene *g*, the weighted linear model

$$ \mathbf{y}_{g} = X \boldsymbol{\beta}_{g} + \sigma_{g} W_{g}^{-\frac{1}{2}} \boldsymbol{\epsilon} $$

can be fitted to log-cpm values

$$\mathbf{y}_{g} = (y_{g11}, \dots, y_{g1n_{1}}, y_{g21}, \dots, y_{g2n_{2}})' $$

with design matrix

$$X = \left(\begin{array} {cc} 1 & 0 \\ \vdots & \vdots \\ 1 & 0 \\ 1 & 1 \\ \vdots & \vdots \\ 1 & 1 \end{array} \right), $$

coefficients vector

$$\boldsymbol{\beta}_{g} = \left(\begin{array}{c} \beta_{g1} \\ \beta_{g2} \end{array} \right), $$

unknown gene-specific standard deviation *σ*_*g*_, associated *voom* precision weights

$$ W_{g} = diag\left(w_{g11}, \cdots, w_{g1n_{1}}, w_{g21}, \cdots, w_{g2n_{2}}\right), $$

and error

$$\boldsymbol{\epsilon} \sim MVN\left(\boldsymbol{0},I_{n_{1}+n_{2}}\right). $$

Thus we could obtain the coefficient estimators

$$ \boldsymbol{\hat{\beta}}_{g} = \left(X^{T} W_{g} X\right)^{-1} X^{T} W_{g} \mathbf{y}_{g} $$

with variance-covariance matrix

$$ Var(\boldsymbol{\hat{\beta}}_{g}) = {\sigma_{g}^{2}} \left(X^{T} W_{g} X\right)^{-1}, $$

where ${\sigma _{g}^{2}}$ is estimated by ${s_{g}^{2}}$

$${s_{g}^{2}} = \frac{\left(\mathbf{y}_{g} - X \boldsymbol{\beta}_{g}\right)' W_{g} \left(\mathbf{y}_{g} - X \boldsymbol{\beta}_{g}\right)}{n-p} $$

with

$$ p = rank(X) = 2. $$

Let *v*_*gk*_ be the *k*th diagonal element of (*X*^*T*^*W*_*g*_*X*)^−1^, where

$$\begin{array}{*{20}l} & \left(X^{T} W_{g} X\right)^{-1} \\ &\quad= \left(\begin{array}{cc} \sum_{i=1}^{2} \sum_{j=1}^{n_{i}} w_{gij} & \sum_{j=1}^{n_{2}} w_{g2j} \\ \sum_{j=1}^{n_{2}} w_{g2j} & \sum_{j=1}^{n_{2}} w_{g2j} \end{array} \right)^{-1} \\ &\quad= \frac{\left(\begin{array}{cc} \sum_{j=1}^{n_{2}} w_{g2j}& -\sum_{j=1}^{n_{2}} w_{g2j}\\ -\sum_{j=1}^{n_{2}} w_{g2j} & \sum_{i=1}^{2} \sum_{j=1}^{n_{i}} w_{gij} \end{array} \right)}{\sum_{j=1}^{n_{1}} w_{g1j} \sum_{j=1}^{n_{2}} w_{g2j}}. \end{array} $$

Under the assumptions as made in Smyth (2004),

$$ \hat{\beta}_{gk} | \beta_{gk}, {\sigma_{g}^{2}} \sim N\left(\beta_{gk}, v_{gk} {\sigma_{g}^{2}}\right) $$

and

$$ {s_{g}^{2}}| {\sigma_{g}^{2}} \sim \frac{{\sigma_{g}^{2}}}{d_{g}} \chi_{d_{g}}^{2}, $$

where *d*_*g*_ is the residual degrees of freedom for the linear model of gene *g*, the ordinary *t*-test statistic will be

$$ t_{gk} = \frac{\hat{\beta}_{gk}}{s_{g} \sqrt{v_{gk}}}, $$

which follows an approximate *t*-distribution with *d*_*g*_ degrees of freedom.

Assuming equal variance between treatment and control group, then the statistic for testing

$$ {H_{0}^{g}}: \mu_{g1} = \mu_{g2} \hspace{3mm} vs. \hspace{3mm} {H_{1}^{g}}: \mu_{g1} \neq \mu_{g2} $$

for the *g*th gene is

$$\begin{array}{*{20}l} T_{g} &= \frac{\hat{\beta}_{g2}}{S.E.(\hat{\beta}_{g2})} \\ &= \frac{\hat{\beta}_{g2}}{s_{g} \sqrt{v_{g2}}} \\ &= \frac{\hat{\beta}_{g2}}{s_{g} \sqrt{\frac{\sum_{i=1}^{2} \sum_{j=1}^{n_{i}} w_{gij} }{\sum_{j=1}^{n_{1}} w_{g1j} \sum_{j=1}^{n_{2}} w_{g2j}}}} \\ &= \frac{\hat{\beta}_{g2} \sqrt{\frac{1}{n_{1}}+\frac{1}{n_{2}}} \sqrt{\frac{\sum_{j=1}^{n_{1}} w_{g1j} \sum_{j=1}^{n_{2}} w_{g2j}}{\sum_{i=1}^{2} \sum_{j=1}^{n_{i}} w_{gij}}}}{s_{g}\sqrt{\frac{1}{n_{1}}+\frac{1}{n_{2}}}}\\ &= \frac{\hat{\beta}_{g2} \sqrt{\frac{\sum_{j=1}^{n_{1}} w_{g1j}/n_{1} \sum_{j=1}^{n_{2}} w_{g2j}/n_{2}}{\sum_{i=1}^{2} \sum_{j=1}^{n_{i}} w_{gij}/(n_{1}+n_{2})}}}{s_{g}\sqrt{\frac{1}{n_{1}}+\frac{1}{n_{2}}}} \\ &= \frac{\hat{\beta}_{g2} \sqrt{\frac{\bar{w}_{g1\cdot} \bar{w}_{g2\cdot} }{\bar{w}_{g\cdot \cdot} }} }{s_{g}\sqrt{\frac{1}{n_{1}}+\frac{1}{n_{2}}}}\\ &\equiv \frac{\Delta_{g}}{s_{g}\sqrt{\frac{1}{n_{1}}+\frac{1}{n_{2}}}}, \end{array} $$

where

$$\Delta_{g} \equiv \hat{\beta}_{g2} \sqrt{\frac{\bar{w}_{g1\cdot} \bar{w}_{g2\cdot} }{\bar{w}_{g\cdot \cdot}}} $$

with $\bar {w}_{g1\cdot } = \frac {1}{n_{1}} \sum _{j=1}^{n_{1}} w_{g1j}$, $\bar {w}_{g2\cdot } = \frac {1}{n_{2}} \sum _{j=1}^{n_{2}} w_{g2j}$ and $\bar {w}_{g\cdot \cdot } = \frac {1}{n_{1}+n_{2}} \sum _{i=1}^{2} \sum _{j=1}^{n_{i}} w_{gij}$.

### Appendix B: Choice of rejection region *Γ* satisfying formula (1)

For the two-sample comparison with *t*-test statistics *T*_*g*_ as in Eq. (3), we assume as in LH method that the effect size follows a normal distribution

$$ \Delta_{g} \sim N\left(\mu_{\Delta}, \sigma_{\Delta}^{2}\right), $$

and the variance of log-cpm values for each gene follows an inverse gamma distribution

$$ {\sigma_{g}^{2}} \sim Inv-Gamma(a,b) $$

with mean $\frac {b}{a-1}$, then formula (1) becomes

(5) $$ {{}\begin{aligned} & \frac{\alpha}{1-\alpha} \frac{1-\pi_{0}}{\pi_{0}} \\ \geq &\,\, \frac{Pr(T \in \Gamma | H = 0)}{Pr(T \in \Gamma | H = 1)} \\ = &\,\, \frac{Pr(T \in \Gamma|H = 0) }{\int \int Pr(T \in \Gamma|H = 1, \Delta_{g}, \sigma_{g}) \pi_{1}(\Delta_{g}) \pi_{2}(\sigma_{g}) d\Delta_{g} d\sigma_{g}} \\ = &\,\, \frac{Pr(|T_{g}| > c | H=0)}{\int \int Pr(|T_{g}| > c | H=1, \Delta_{g}, \sigma_{g}) \pi_{1}(\Delta_{g}) \pi_{2}(\sigma_{g}) d\Delta_{g} d\sigma_{g}},  \end{aligned}}  $$

where *π*_1_(*Δ*_*g*_) and *π*_2_(*σ*_*g*_) denote the probability distribution function (p.d.f.) of *Δ*_*g*_ and *σ*_*g*_ respectively. The numerator in (5) equals

$$2 \cdot T_{n_{1}+n_{2}-2}(-c), $$

where *T*_*d*_(·) denotes the cumulative distribution function (c.d.f.) of a central *t*-distribution with *d* degrees of freedom, and the denominator in (5) equals

(6) $$\begin{array}{*{20}l}  1 &- \int\int T_{n_{1}+n_{2}-2}\left(c|\theta_{g}\right)\pi_{1}(\Delta_{g}) \pi_{2}(\sigma_{g}) d\Delta_{g} d\sigma_{g} \\ &+\int\int T_{n_{1}+n_{2}-2}\left(-c|\theta_{g}\right) \pi_{1}(\Delta_{g}) \pi_{2}(\sigma_{g}) d\Delta_{g} d\sigma_{g}.  \end{array} $$

Here, *T*_*d*_(·|*θ*_*g*_) denotes the c.d.f. of a non-central *t*-distribution with *d* degrees of freedom and non-centrality parameter

$$ \theta_{g} = \frac{\Delta_{g}}{\sigma_{g}\sqrt{\frac{1}{n_{1}} + \frac{1}{n_{2}}}}. $$

The integration in (6) with respect to *Δ*_*g*_ could be avoided through mathematical derivation, and the integration with respect to *σ*_*g*_ is approximated using static quadrature rules, which allows a stable calculation to get the root of *c*. Details of derivation could be found in Appendix B of [14].

Once the choice of *c* has been made for each sample size, power would be calculated accordingly by integrating over the prior distributions on effect size and residual variance. Hence based on the desired power, sample size is finally determined.

## Additional file

Additional file 1 This excel file contains comparison of resulting sample size and power between Li et al.’s method [19] and our proposed method for simulation 1, with parameter settings from Table 1 in [19]. The results are obtained under *m*=200, with Li’s result in the first row from each parameter setting, and our result in the second row. (XLS 49.2 kb)
